# VIRTUAL REALITY AND OLDER ADULTS: A USABILITY STUDY

**DOI:** 10.1093/geroni/igz038.897

**Published:** 2019-11-08

**Authors:** Julie A Brown

**Affiliations:** Ohio University, Athens, Ohio, United States

## Abstract

Within the past two years there has been a very small but growing number of scholarly articles that highlight the potential benefit of mobile Virtual Reality (VR) platforms among older adult populations. Yet, it is critical to assess older adult user needs and preferences, as well as ethical considerations, before utilizing VR in applied contexts. This pilot study investigated perceptions of VR use and its potential application by individually interviewing ten community-dwelling older adults (ages 63 to 89) both before and after trying the Samsung Gear VR and followed with two focus group discussions. Themes identified from the transcripts include 1) usability, 2) video subject matter preferences, and 3) implications with use. These themes highlighted both the challenges and opportunities of VR use among a wide range of older populations, and provided greater insight with its exploration and application in future studies, particularly with individuals with functional limitations.

